# Proinsulin Promotes Self-Renewal of a Hematopoietic Progenitor Cell Line In Vitro

**DOI:** 10.1155/2017/5649191

**Published:** 2017-07-03

**Authors:** Yuewen Han, Tingting Liu, Yunding Zou, Ling Ji, Yuanyuan Li, Jing Li, Jing Wang, Guopin Chen, Jieping Chen, Liang Chen, Zhijia Ye

**Affiliations:** ^1^Institute of Tropical Medicine, Third Military Medical University, Chongqing, China; ^2^Bioengineering College, Chongqing University, Chongqing, China; ^3^Xi'an Center for Disease Control and Prevention, Xi'an, China; ^4^Department of Hematology, Southwest Hospital, Chongqing, China; ^5^Biomedical Analysis Center, Third Military Medical University, Chongqing, China; ^6^Department of Plastic and Aesthetic Surgery, Southwest Hospital, Chongqing, China

## Abstract

The objective of this study was to assess the effects of exogenously expressed proinsulin on the biological characters of a hematopoietic stem cell line (HSC) and erythroid myeloid lymphoid (EML) cells and explore new strategies for cell therapy for type I diabetes. EML cells were transduced with lentivirus particles carrying the human proinsulin (proINS) gene. The positive transduced cells were selected based on green fluorescence protein (GFP) positivity and puromycin resistance. Overexpression of proINS was confirmed via real-time PCR and Western blotting. The functional activity of the human proINS secreted by EML cells was elucidated by analyzing the activation of insulin receptor and its downstream signaling. Pro-INS + EML cells were able to prime the phosphorylation of insulin receptor as well as induce the expression of downstream genes of insulin receptor. Furthermore, Wnt3a can significantly promote self-renewal of Pro-INS + EML cells. However, we did not observe significant changes in the proliferation and differentiation of INS + EML cells, compared to the control EML cells. Our results might be useful for developing a new therapy for diabetes mellitus.

## 1. Introduction

Diabetes mellitus, a widespread metabolic disorder, is a global epidemic with a continuous, steep increase in global prevalence and incidence [1]. There are two forms of diabetes mellitus: type I, when the pancreas does not produce enough insulin [2], and type II, when the body cannot effectively use the produced insulin [3]. In both cases, chronic high blood glucose levels cause severe complications, including macroangiopathy and microangiopathy, leading to blindness, chronic renal insufficiency, central and peripheral neuropathy, and cardiovascular disease [3].

Conventional therapy for diabetes based on exogenous insulin or oral agents may control and delay, but not prevent, disease-related complications [2, 4]. Despite the promising outcomes observed with islet transplantation and progress in immunomodulatory therapies, the need for an effective cell replacement strategy for diabetes remains. Especially, stem cell-based strategies represent significant therapeutic potential owing to both the intrinsic regenerative capacity and the immunomodulatory potential of stem cells [5]. In the past years, substitution of failed *β*-cells by embryonic stem cells (ESCs) or induced pluripotent stem cells (iPSCs) raised hope for diabetes cure [5, 6]. Some successful cases showed that procedures that promote ESC differentiation into insulin-secreting cells have been developed for animal models [5]. However, the risk of immune rejection and lack of efficacy in the conversion of ESCs into *β*-cells are yet to be tackled. As iPSCs are generated from autologous cells, graft-directed immune destruction is likely circumvented by reprogramming the patient's specific fibroblast into type 1 diabetes patient-specific iPSCs [7]; however, generating human iPSCs suitable for clinical application is still a long way ahead. In addition, formation of *β*-cells from ESCs as well as iPSCs is technically problematic and quite expensive [5].

Hematopoietic stem cells (HSCs) are multipotent stem cells residing in the bone marrow and umbilical cord blood, capable of differentiating into different lineages of blood cells [8]. More interestingly, HSCs have the potential to correct and/or reeducate the immune aberration that promotes autoimmune attacks against host tissue. Therefore, HSC transplantation has been used as a therapeutic strategy for different types of autoimmune diseases and applied to type 1 diabetes patients in a minimum of two clinical trials with promising results [9, 10]. Previous studies show that insulin can be an important factor for HSC modulation. Interactions between the insulin signaling pathway and the canonical Wnt signaling pathway can promote relatively stable self-renewal of HSCs [11, 12]. For example, insulin activates PI3K/Akt pathway, inhibits apoptosis of HSCs, and promotes expansion of HSCs. Combined treatment with insulin and CHIR99021, a GSK inhibitor, can support efficient HSC expansion in vitro [12]. However, whether and how the exogenously expressed proinsulin affects the biological characteristics of HSCs remains unknown. In this study, we report the effects of exogenous proinsulin on the self-renewal and differentiation of EML cells, which had been demonstrated to be a unique model very similar to wild-type HSCs [13].

## 2. Materials and Methods

### 2.1. Cell Lines and Culture

Erythroid myeloid lymphoid (EML) cells were cultured with Iscove's modified Dulbecco's medium (IMDM), supplemented with 10% fetal bovine serum (FBS) (Hyclone, Logan, UT, USA), 15% conditional medium of BHK-21 cells (containing stem cell factor), 100 U/ml penicillin, and 100 *μ*g/ml streptomycin. To construct a proinsulin (Pro-INS) overexpressing cell line, the human Pro-INS open reading frame (GenBank number, NM_000207) was synthesized and recombined into the lentivirus vector pCDH-CMV-MCS-EF1-GFP-T2A-Puro. The pCDH-Pro-INS or pCDH plasmids were cotransfected with the packaging plasmids (psPAX2 and pMG2.D) into 293FT cells (around 70–80% confluency). The medium was changed after 12 h, and the virus-containing supernatant was harvested 48 h later. The resulting virus-containing supernatant was then used to infect EML cells, and the positive cells were screened using the dual markers for GFP and puromycin.

### 2.2. Quantitative Real-Time Reverse-Transcription Polymerase Chain Reaction (RT-qPCR)

Total RNA was isolated from the cultured cells using the RNeasy Mini Kit (QIAGEN) as per the manufacturer's protocol. Total RNA was reverse-transcribed using the M-MLV RTase cDNA Synthesis Kit (TaKaRa). Real-time quantitative RT-PCR was performed with the iQ SYBR Green Supermix (Bio-Rad) and normalized against GAPDH expression. The primers are as follows: insulin, 5′-CAGCCGCAGCCTTTGT-3′ and 5′-TTCCACAATGCCACGCT-3′; insulin receptor (IR), 5′-CGAGTGCCCGTCTGGCTATA-3′ and 5′-GGCAGGGTCCCAGACATG-3′; GLUT1, 5′-GAGCATCTTCGAGAAGGCAGGTGT-3′ and 5′-GGCCACAATGAACCATGGAATA-3′; and GAPDH, 5′-TGTGTCCGTCGTGGATCTGA-3′ and 5′-CCTGCTTCACCACCTTCTTGAT-3′.

### 2.3. Western Blotting

For Western blotting, the cells were collected and lysed in radioimmunoprecipitation assay (RIPA) buffer with protease inhibitors on ice. Approximately 30 *μ*g of the sample was boiled for 5 min, separated using 10% SDS-PAGE, and then transferred to polyvinylidene fluoride (PVDF) membranes (Millipore, Billerica, MA, USA). After incubation with a blocking buffer, the membranes were incubated with mouse anti-insulin (Cell Signaling Technology, Danvers, MA, USA), mouse anti-insulin receptor *β* (Cell Signaling), rabbit anti-phospho-IGF-I receptor *β* (Tyr1131) (Cell Signaling), mouse anti-phospho-Akt (Thr308) (Abcam), and rabbit anti-*β*-actin (Cell Signaling) primary antibodies and horseradish peroxidase-conjugated secondary antibodies, including horse anti-mouse and goat anti-rabbit antibodies (Cell Signaling).

### 2.4. Flow Cytometry

Cells were harvested, and single-cell suspensions were prepared in cool phosphate-buffered saline (PBS) containing 0.5% bovine serum albumin (BSA) and stained with fluorescein isothiocyanate (FITC) anti-CD34 (BD Biosciences, California, USA), FITC Annexin V (BioLegend, CA, USA), allophycocyanin (APC) anti-B220 (eBioscience, CA, USA), phycoerythrin (PE) anti-Sca-1 (eBioscience), and PE-CD11b (eBioscience). All samples were analyzed using FACS Aria I (BD Biosciences), and the data were analyzed using the FlowJo software (Tree Star Inc.).

### 2.5. 5-Bromo-2'-deoxyuridine (Brdu) Incorporation Assay

The cells were pulsed with 20 *μ*M BrdU, maintained in the medium for 30 min, and collected and fixed in cold 70% ethanol. The fixed cells were treated with 4 N HCl, neutralized with 0.1 M borax, and washed in PBS containing 0.05% BSA. Next, the cells were incubated with anti-BrdU antibody (BD Biosciences) in 0.5% BSA, followed by incubation with FITC-conjugated anti-mouse secondary antibody (Sigma) in PBS with 0.5% Tween-20. The cells were then resuspended in PBS containing propidium iodide (PI) and RNase A and analyzed via fluorescence-activated cell sorting (FACS).

### 2.6. CFU-Based Assay

To assess their differentiation capacity, total EML/control and EML/proINS cells were plated in 1 ml of MethoCult H4035 Optimum without EPO (Stemcell Technologies, Vancouver, Canada) in 6-well plates (10^3^ cells/well) in replicates. The medium was supplemented with 8 U/ml recombinant human erythropoietin (PeproTech, NJ, USA) for the generation of erythroid burst-forming units (BFU-E). The cultures were maintained at 37°C, 5% CO_2_ for 10–14 days. The BFU-E, CFU-GM, and CFU-Meg colonies were counted after 10–14 days of plating.

## 3. Results

### 3.1. Exogenous Expression of Proinsulin in EML Cells

**Figure 1:**
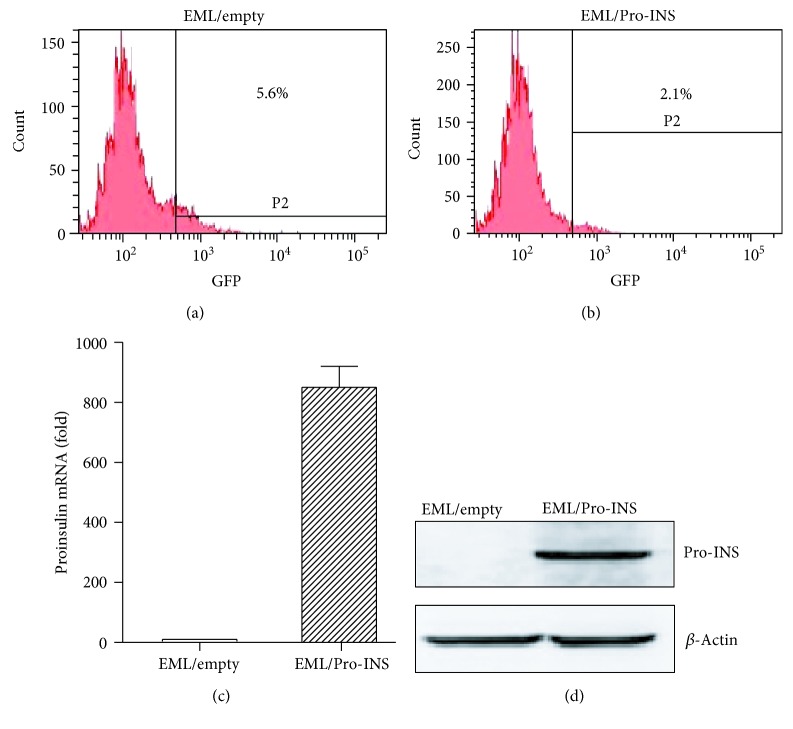
Overexpressed proinsulin identified in EML cells. (a), (b) GFP-positive cells were detected via FACS in EML cells transduced by lentivirus particles carrying empty vector (empty) or proinsulin gene (Pro-INS), respectively. (c) Overexpressed proinsulin mRNA was detected using real-time PCR in EML cells transduced by LV-V carrying the proinsulin gene. (d) Proinsulin protein was detected via Western blotting in EML cells transduced by LV-V carrying the proinsulin gene.

To construct stable EML cell lines expressing proinsulin, we cloned INS cDNA into the lentivirus vector pCDH-CMV-MCS-EF1-GFP-T2A-Puro, generated pseudoviral particles carrying INS, and transduced EML cells with the lentivirus particles at an ROI of 100 : 1. Because the lentivirus vector contains both GFP gene and puromycin resistance gene, we used both markers for evaluation and selection of transductants. On day 1 after infection, ~5% GFP-positive EML cells were detected (Figures 1(a) and 1(b)). EML cells with stable GFP expression were obtained on day 10 after puromycin selection. Unlike the control EML cells transduced with lentivirus particles carrying empty vector (EML/empty), EML cells transduced with lentivirus particles carrying the proinsulin gene (EML/Pro-INS) were detected based on the expression of proinsulin. INS mRNA and proinsulin protein were verified using real-time quantitative PCR (Figure 1(c)) and Western blotting (Figure 1(d)), respectively. Approximately 10 pg/ml proinsulin was detected in the supernatant of the culture medium of EML/Pro-INS cells via ELISA analysis (data not shown).

### 3.2. Secreted Proinsulin Activated the Insulin Receptor Pathway

**Figure 2:**
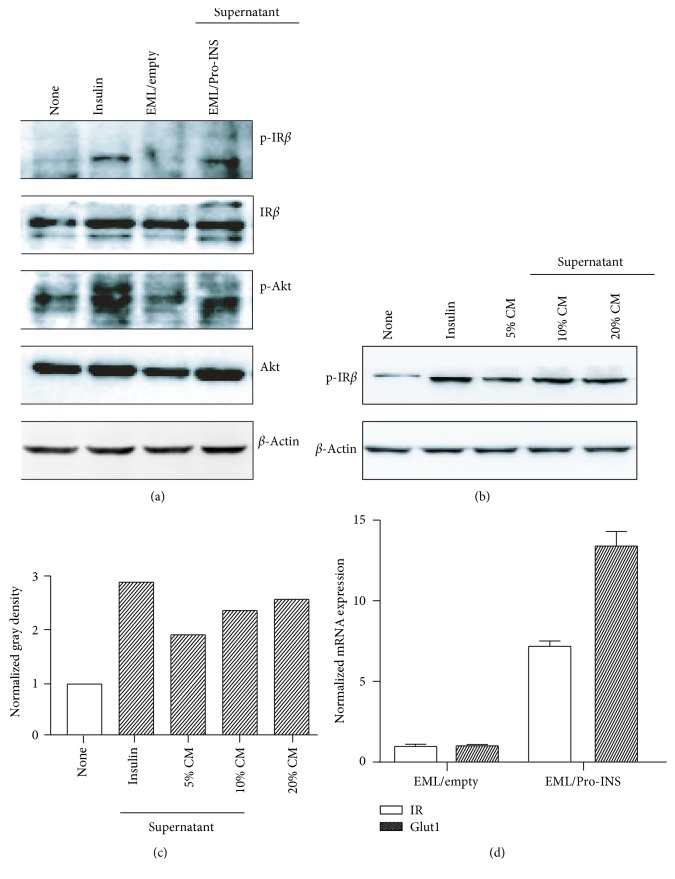
Signaling pathway mediated by the insulin receptor (IR) activated by proinsulin. (a) Serum-starved MEF cells (for 12 h) were stimulated with insulin, 20% CM of EML/empty, and 20% CM of EML/Pro-INS, respectively, for 10 min. Then, the cells were harvested and lysed in RIPA buffer. The phosphorylated *β*-subunit of IR (IR*β*) and phosphorylated Akt were detected via Western blotting. (b) Serum-starved MEF cells (for 12 h) were exposed to insulin for 10 min and increasing doses of CM of EML/Pro-INS, respectively. The phosphorylated IR*β* was detected via Western blotting. The ratio of the gray density of phosphor-IR*β* versus total IR*β* was measured and calculated. (c), (d) The levels of IR and Glut1 mRNA expression in MEF cells stimulated with 20% CM of EML/empty and 20% CM of EML/Pro-INS, respectively, were detected using real-time PCR.

Proinsulin is a prohormone with low metabolic activity compared to mature insulin. Proinsulin differentially binds to and activates the two insulin receptor (IR) isoforms, with higher affinity for IR-A than IR-B [14], and then the activated *β*-subunits of IR consequently activate the downstream molecules and turn on signaling pathways, including Src/PI3K/Akt [15]. To determine whether the proinsulin secreted by EML/Pro-INS can activate the IR-mediated signaling pathway, we analyzed the phosphorylation of *β*-subunits of IR and the phosphorylation of Akt in mouse embryonic fibroblast (MEF) cells stimulated with the CM of EML/Pro-INS. We found that the phosphorylation levels of IR and Akt significantly increased in the MEF cells treated with 20% CM of EML/Pro-INS cells (Figure 2(a)). The phosphorylation levels of IR positively correlated with the doses of the added CM concentration (Figures 2(b) and 2(c)). We also assayed the gene expression levels of IR and Glut1, which are involved in the regulation of glucose metabolism, and found that the expression levels of IR and Glut1 significantly increased in MEF cells (Figure 2(d)).

### 3.3. Exogenously Expressed Proinsulin Had No Significant Influence on the Proliferation and Differentiation of Hematopoietic Progenitor Cells

**Figure 3:**
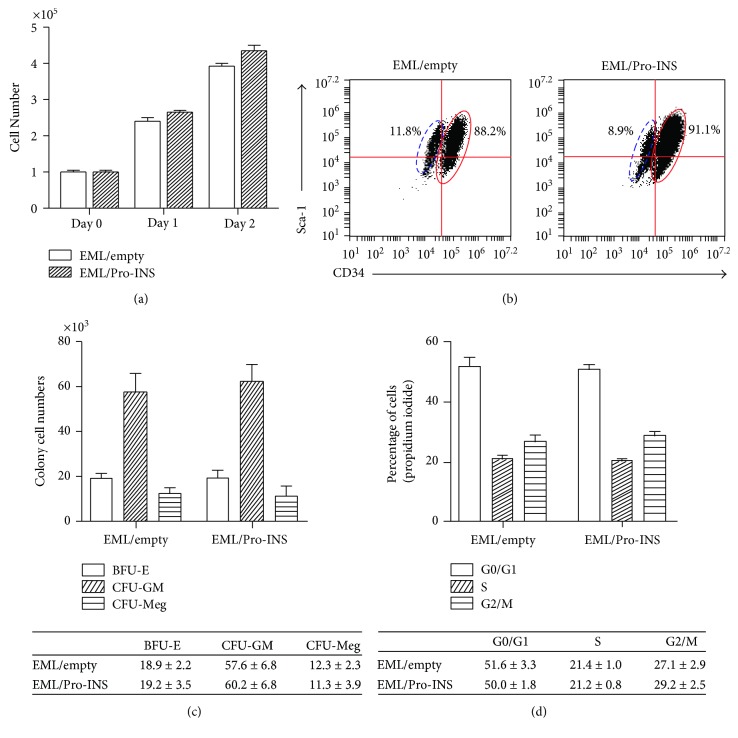
Effects of exogenous expression of proinsulin on the proliferation, self-renewal, and differentiation of EML cells. (a) EML/empty cells and EML/Pro-INS cells were cultured in fresh EML medium at an initial concentration of 1 × 10^5^. Cell numbers were determined using trypan blue on days 1 and 2. (b) The distribution of the CD34^+^Sca-1hi and CD34^−^Sca-1low subpopulations in EML/empty cells and EML/Pro-INS cells was studied via FACS. (c) CFU-based assay of the differentiation capacity of EML/empty cells and EML/Pro-INS cells into erythroid, granulocyte-macrophage, and megakaryocytic lineages. (d) Analysis of the cell cycle profiles of EML/empty cells and EML/Pro-INS cells by propidium iodide staining.

Proinsulin inhibits apoptosis and promotes the survival of neuroepithelial cells or neurons during early neural development [16]. In order to assess whether proinsulin affects the proliferation of EML cells, we measured the proliferation capacity of EML/Pro-INS cells by Brdu incorporation assay. We found no significant difference in Brdu incorporation between EML/Pro-INS cells and control cells (Table 1, Figure 3(a)). In contrast, the population of Annexin V-positive cells in EML/proINS is lesser than that in EML/control cells (Table 1). Thus, the expressed proinsulin prohibits the apoptosis of EML cells.

Previously, we found that CD34^+^Sca-1hi EML cells autonomously differentiated into CD34^−^Sca-1low after being cultured in SCF medium [17]. In this study, we addressed the two questions: whether and how the exogenously expressed proinsulin influences the distribution of CD34^+^Sca-1hi and CD34^+^Sca-1low subpopulations in EML cells. Interestingly, we did not observe a significant difference in the distribution of CD34^+^Sca-1hi and CD34^−^Sca-1low in both EML/Pro-INS and EML/empty cells (Figure 3(b)). This indicated that proinsulin had no effect on self-renewal and differentiation of population of CD34^+^Sca-1hi.

EML cell line has a well-documented multilineage differentiation capacity and can differentiate into erythroid, myeloid, and lymphoid progenitors in the presence of SCF [13, 18]. In this study, we studied the influence of expressed proinsulin on lineage differentiation by checking CFU formation. We observed very similar patterns of BFU-E and CFU-GM and CFU-Meg in both EML/Pro-INS cells and EML/empty cells (Figure 3(c)). These results indicated that there were no significant effects of the exogenously expressed proinsulin on the lineage-biased differentiation of EML cells. The data obtained for cell cycle profile assay involving PI staining showed that there were significantly different population sizes in the G0/G1 and S/G2/M phases in EML/proINS, compared to EML/empty (Figure 3(d)). Cumulatively, exogenously expressed proinsulin did not affect the ability of proliferation and lineage differentiation of EML cells.

### 3.4. Effects of the Interaction between Wnt3a and Proinsulin on Self-Renewal and Differentiation of Hematopoietic Progenitor Cells

**Figure 4:**
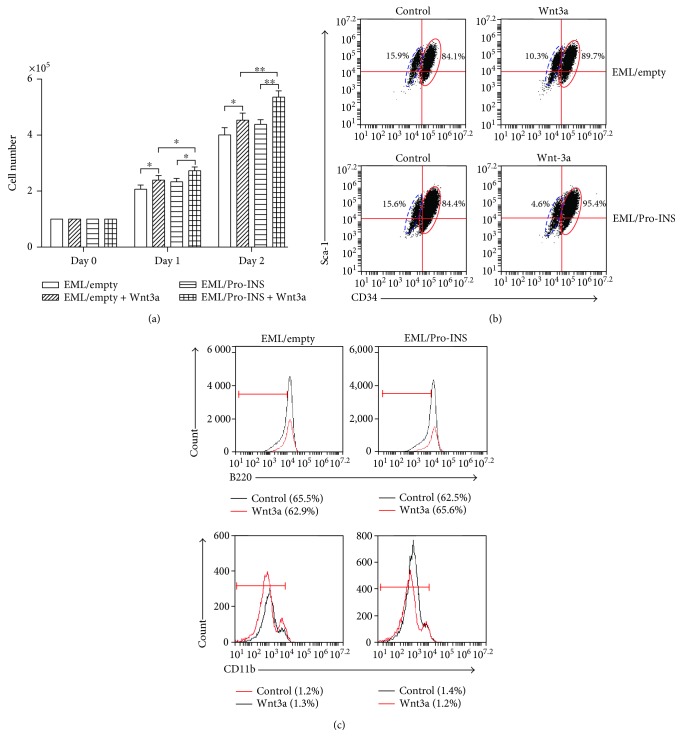
Synergistic effects of proinsulin and Wnt3a on the proliferation, self-renewal, and differentiation of EML cells. (a) EML/empty cells and EML/Pro-INS cells were cultured in fresh EML medium with or without Wnt3a (50 ng/ml) at an initial concentration of 1 × 10^5^ (day 0). The total number of living cells assessed via trypan blue staining was counted on days 1 and 2. (b) The distribution of CD34^+^Sca-1hi and CD34^−^Sca-1low subpopulations was detected via FACS. (c) The percentage of lineage marks of B220 and CD11b was tested in the above cells via FACS. ^∗^*p* < 0.05 and ^∗∗^*p* < 0.001.

Wnt signaling is required for normal self-renewal and other functions of HSCs. The synergistic effects of Wnt/*β*-catenin signaling and PI3K/Akt signaling on the self-renewal and expansion of normal HSCs were observed in vitro and in vivo [11, 12]. The combination of Wnt signaling activator (Wnt3a and CHIR99021) and insulin was shown to completely instead of the conventional cytokines and growth factors that are required for the expansion of normal HSCs in vitro. To verify whether proinsulins like insulin can cooperate with Wnt signaling to regulate self-renewal and differentiation of EML cells, we assayed the proliferation and differentiation of INS + EML cells treated by Wnt3a, the typical activator of Wnt signaling. We found that the significantly increased proliferation induced by Wnt3a was observed only in INS + EML cells, but not in the mock EML cells. Wnt3a not only decreased the population of Annexin V-positive INS + EML cells but also increased Brdu incorporation (Table 1). Those data indicated that the synergy of proinsulin and Wnt3a promoted the proliferation of EML cells by inhibiting cell apoptosis and enhancing cell growth (Figures 4(a) and 4(b)).

Similarly, we assayed the effects of Wnt3a on proliferative self-renewal and lineage differentiations of INS + EML cells. We found that the ratio of subpopulation of CD34^+^Sca-1hi versus that of CD34^−^Sca-1low increased on Wnt3a stimulation in EML/proINS compared to EML/empty (Figure 4(c)). Meanwhile, we analyzed the lineage differentiation in EML/proINS by detecting lineage markers, including B220 and CD11b (Figure 4(c)). Wnt3a treatment did not have a significant effect on lineage differentiation in EML/Pro-INS compared to control EML cells. These results indicated that the combination of proinsulin and Wnt3a promoted self-renewal of the hematopoietic progenitors but did not inhibit their lineage differentiation.

## 4. Discussion

As a prohormone, proinsulin had been shown to have low metabolic activity compared to mature insulin. However, proinsulin can promote other effects in the regulation of growth and development in the embryonic stages [19–21]. Proinsulin is expressed in the developing chicken retina; it plays an autocrine/paracrine stimulatory role during neurogenesis and prevents cell death of embryos subjected to growth factor deprivation. Proinsulin was expressed in the rat yolk sac, where hematopoietic stem cells emerged in the late gestational stages [22]. The effects of proinsulin on the self-renewal and differentiation of HSCs remain unknown.

The EML cell line, generated by a transducing retrovirus carrying dominant mutated amino acid receptor, represents a good model for studying hematopoietic stem cells. EML cells are expanded from a single-cell clone, but contain heterogeneous subsets, displaying different phenotypes and functions. In our previous study, we isolated and identified two subpopulations, Lin-c-kit + CD34^+^Sca-1hi and Lin-CD34^−^Sca-1low, from parental EML cells. We found that the CD34^+^Sca-1hi cells were able to undergo self-renewal and differentiate into CD34^−^Sca-1low cells as well. We assumed that CD34^+^ cells represented the phenotype of the original EML clone. In this study, we evaluated the influence of exogenously expressed proinsulin on the proliferation of total EML cells and distribution of subpopulations. We found that the exogenous expression of proinsulin enhanced the proliferation of total EML by inhibiting apoptosis. On the other hand, we did not observe significant changes in the distribution of subpopulations. These results indicated that proinsulin can be a potentially useful reagent for promoting the proliferation of HSCs without influencing. This observation corresponds to some recent reports on the effect of matured insulin on mouse wild-type HSCs [11].

Wnt signaling has been demonstrated to play a significant role in regulating self-renewal and differentiation of HSCs [23]. Various interactions of Wnt signaling and other signaling pathways coordinate the expansion and differentiation of HSCs as well as the progenitors of different stages. Evidence supports the idea that mTOR signal pathway and Wnt signal pathway synergistically increased self-renewal of human cord blood HSCs and mouse bone marrow HSCs [12]. In this study, we observed a significant synergistic nature between the exogenously expressed proinsulin and Wnt3a stimulation on promoting the self-renewal ability of CD34^+^ cells and repressing differentiation of CD34^+^ cells. Insulin activates mTORC1/2 and its downstream molecules via IRs and regulates the metabolism and proliferation of targeted cells. Malaguarnera et al. reported that proinsulin promoted the phosphorylation of p70S6K and Erk to an extent similar to insulin [14]. Furthermore, the group found that proinsulin played almost as equipotent roles as insulin in inducing cell proliferation and migration in cells expressing IR-A, although the metabolic activity was lower [14].

Proinsulin acts similar to insulin in reducing blood glucose [24]. Intravenous administration of adenovirus carrying proinsulin reversed high blood glucose levels in a mouse diabetes model [25]. In this study, we found that exogenously expressed proinsulin stimulated the phosphorylation of IR and induced the expression of Glut1, which mediates glucose uptake by the target cells. We observed that proinsulin induced similar levels of phosphorylation of IR than mature insulin did. The levels of phosphorylated IR induced by proinsulin positively correlated with the dose of proinsulin. On binding with IR, insulin activates several signaling pathways and induces expression of target genes, which are involved in the regulation of glucose metabolism. As one of the target genes of the signaling pathways mediated by insulin, Glut1 plays a critical role in enhancing glucose uptake. Our results indicated that proinsulin, a kind of prohormone, is involved not only in regulating the growth and differentiation of HSCs but also in glucose metabolism. It is important to fully elucidate the physiological functions of the exogenously expressed proinsulin in regulating HSCs as well as its potential pharmacological application in diabetes therapy.

## Figures and Tables

**Table 1:** Proliferation and apoptosis of EML/empty and EML/Pro-INS.

	EML/empty		EML/Pro-INS	
	Control	Wnt3a	Control	Wnt3a
BrdU + %	56.3 ± 5.7	65.5 ± 5.9	58.9 ± 5.2	68.8 ± 6.4
Annexin V + %	15.5 ± 1.8	14.9 ± 1.6	11.9 ± 1.2	10.2 ± 1.8
